# The complete chloroplast genome of *Eremurus zoae* Vved. (Asphodelaceae), an endemic species of Kyrgyz Republic

**DOI:** 10.1080/23802359.2024.2336003

**Published:** 2024-04-03

**Authors:** Ju Eun Jang, Hyeon Jin Jeong, Aleksey L. Kim, Ye-Rim Choi, Georgii A. Lazkov, Chang-Gee Jang, Hyeok Jae Choi, Hee-Young Gil

**Affiliations:** a Division of Forest Biodiversity, Korea National Arboretum, Pocheon, South Korea; b Institute for Biology, National Academy of Sciences, Bishkek, Kyrgyzstan; c Department of Biology Education, Kongju University, Gongju, South Korea; d Department of Biology & Chemistry, Changwon National University, Changwon, South Korea

**Keywords:** Asphodelaceae, complete chloroplast genome, *Eremurus zoae*, Kyrgyzstan endemic species, phylogenetic analysis

## Abstract

*Eremurus zoae* Vved. 1971 is a perennial herbaceous plant in the family Asphodelaceae and an endemic species of the Kyrgyz Republic; however, its complete chloroplast genome sequence has not been reported. Here, we investigated the complete chloroplast (cp) genome of *E. zoae* using next-generation sequencing. The cp genome was 153,744 bp long, with a large single copy (84,020 bp), a small single copy (16,766 bp), and a pair of inverted repeats (26,479 bp). The genome encodes 132 genes, including 86 protein-coding genes, 38 tRNA genes, and 8 rRNA genes. Phylogenetic analysis revealed that the genus *Eremurus* forms a monophyletic group and *E. zoae* is closely related to *E. chinensis*. This study provides a molecular foundation for future phylogenetic studies of *Eremurus*.

## Introduction

**Figure 1.**
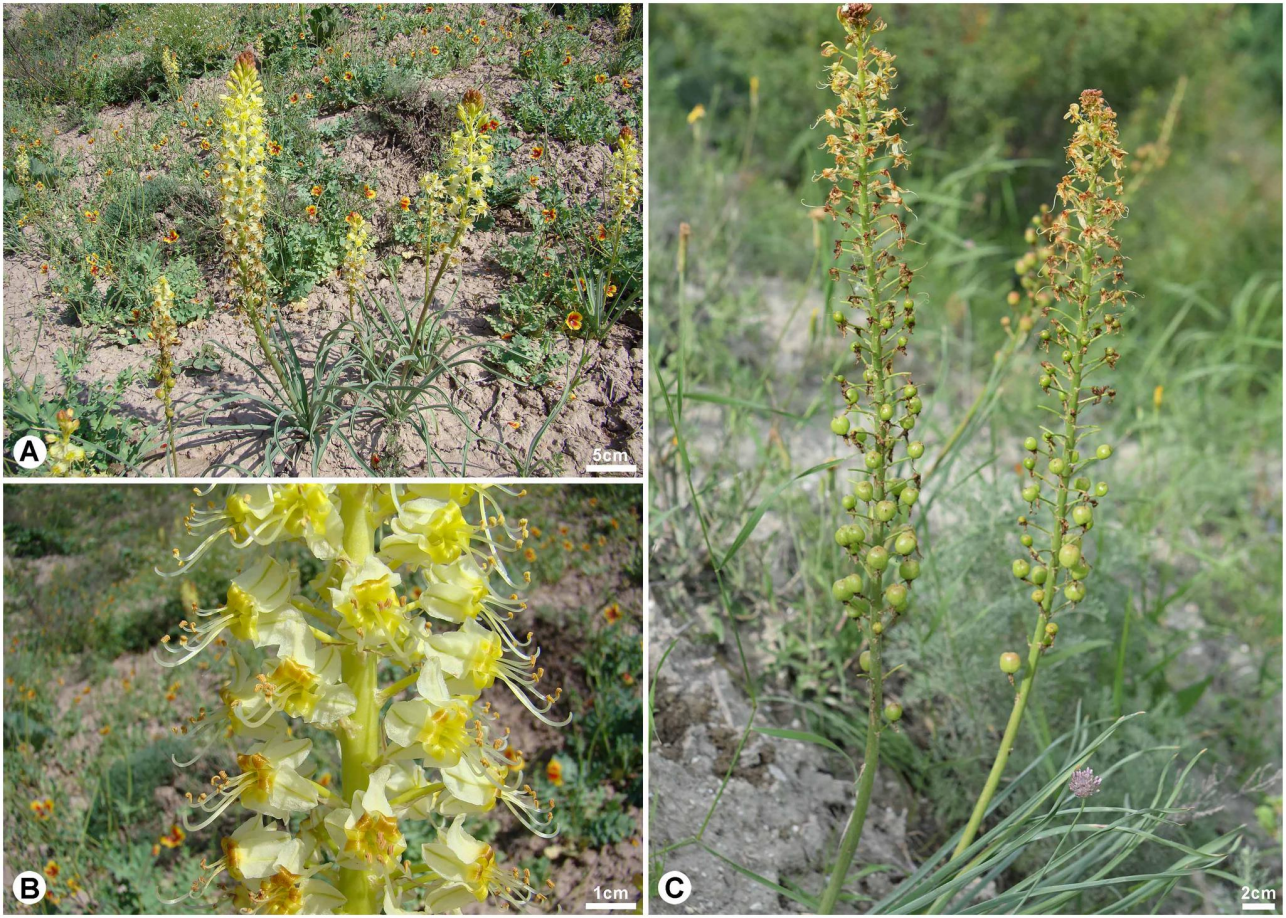
*Eremurus zoae* Vved 1971 (Photographs was taken by Georgy A. Lazkov in Issyk Kul, Kyrgyz Republic). A, Habitat; B, Flower; C, Capsule. *E. zoae* is a perennial herb with a height of 25–40 cm. The flowers are campanulate, with yellow petals that reflex in fruiting. The capsules are globose. The flowering period is from April to May and the fruiting period is from May to June.

*Eremurus* M.Bieb. (Asphodelaceae) is known as foxtail lilies or desert candles, which is a perennial herbaceous plant belonging to the Asphodelaceae family (APG IV 2016; Muhidinov et al. 2020; Farhadi et al. 2023). This genus, which comprises over 50 species, is mainly distributed in Central and Western Asia, with the center of its diversity in Central Asia (Hedge and Wendelbo 1963; Naderi Safar et al. 2009; Farhadi et al. 2023). It is characterized by swollen, fleshy, thick roots; leafless flowering stems; white to pink or yellow flowers; and campanulate or funnel-shaped perianths (Fedchenko 1968; Xinqi et al. 2000; Naderi Safar et al. 2009; Farhadi et al. 2023). Modern pharmacological research has shown that many species in this genus exhibit antibacterial, antimicrobial, antioxidant, anti-inflammatory, antiradical, and anti-glycation activities (Karaman et al. 2011; Zhu et al. 2013). *Eremurus* is also an economically important plant with edible, medicinal, and ornamental uses (Kamenetsky and Rabinowitch 1999; Naderi Safar et al. 2009; Safar et al. 2014; Farhadi et al. 2023). Additionally, several *Eremurus* species contain water-soluble glucomannans and galactomannans in their roots. These substances become sticky when in contact with water and are utilized in the form of glue (Makhmudjanov et al. 2019; Muhidinov et al. 2020). *Eremurus zoae* Vved. 1971, which is endemic to Kyrgyz Republic, can be distinguished from related species by its 25–40 cm tall (Figure 1A), rather short and dense conical-cylindrical raceme with yellow flowers (Figure 1B). It is also often misidentified as *Eremurus luteus* Baker, but this species is clearly different from *E. luteus* by the shape of its capsule (Figure 1C): *E. luteus* has an elongated capsule, whereas *E. zoae* has a globose capsule (Lazkov and Sennikov 2015). This species also faces threats from factors such as overgrazing and plant collection. Consequently, it was classified as vulnerable (VU) in the Red Data Book of the Kyrgyz Republic (2006). Therefore, investigating the genetic diversity and structure of this species to conserve its genetic resources is essential. Although the chloroplast (cp) genome is a very useful genetic tool for inferring phylogenetic relationships, few studies involving the complete cp genome of *Eremurus* have been published (Cho et al. 2015; Song et al. 2022). Here, we report the complete cp genome of *E. zoae* for the first time to clarify the phylogenetic relationships between *E. zoae* and related species and to facilitate further research for a systematic understanding of *Eremurus*.

## Materials and methods

*Eremurus zoae* was collected from Issyk Kul, Kyrgyz Rupublic (latitude 42°27′32.4″, longitude 76°05′42.0″; Figure 1). The voucher specimen was deposited at the herbarium of the Korea National Arboretum (KH, http://www.nature.go.kr/kbi/plant/smpl/KBI_2001_030100.do, Hee-Young Gil, E-mail: warmishe@korea.kr) under the voucher number KZ_160513_403. Fresh leaves were dried using silica gel. DNA was extracted from the silica gel-dried leaves using a DNeasy Plant Mini Kit (Qiagen Inc., Valencia, CA, USA). Next-generation sequencing was conducted on the MiSeq platform (Illumina Inc., Seoul, South Korea) with a 500 bp insert size, and 9,009,222 reads were obtained. We performed *de novo* assembly using GetOrganelle (Jin et al. 2020) with *E. chinensis* as a reference (NC_070056) and conducted the final assembly of the complete cp genome using the Geneious Prime program (Kearse et al. 2012). The cp genome was annotated using GeSeq (Tillich et al. 2017) and the tRNAscan-SE software (Lowe and Chan 2016). A circular map of the *E. zoae* cp genome and cis- and trans-spliced genes was generated using CPGview (Liu et al. 2023). The complete cp genome of *E. zoae* was submitted to GenBank under the accession number OR264467.

To investigate the phylogenetic relationships, we analyzed 17 species together with three outgroup taxa (one Xeronemataceae and two Iridaceae), which were downloaded from NCBI GenBank, except *E. zoae*. For phylogenetic analysis, 82 protein-coding genes were aligned using MAFFT, and the most suitable model for the maximum likelihood (ML) and Bayesian inference (BI) methods was determined using ModelFinder in the PhyloSuite software (Katoh and Standley 2013; Kalyaanamoorthy et al. 2017; Zhang et al. 2020). The ML tree was constructed using IQ-TREE under the GTR + R3 + F model with 5,000 ultrafast bootstraps, whereas the BI tree was inferred using MrBayes 3.2.6 under the GTR + F + I + G4 model (two parallel runs, 2,000,000 generations), in which the initial 25% of the sampled data were discarded as burn-in (Guindon et al. 2010; Ronquist et al. 2012; Minh et al. 2013; Nguyen et al. 2015; Zhang et al. 2020).

## Results and discussion

**Figure 2.**
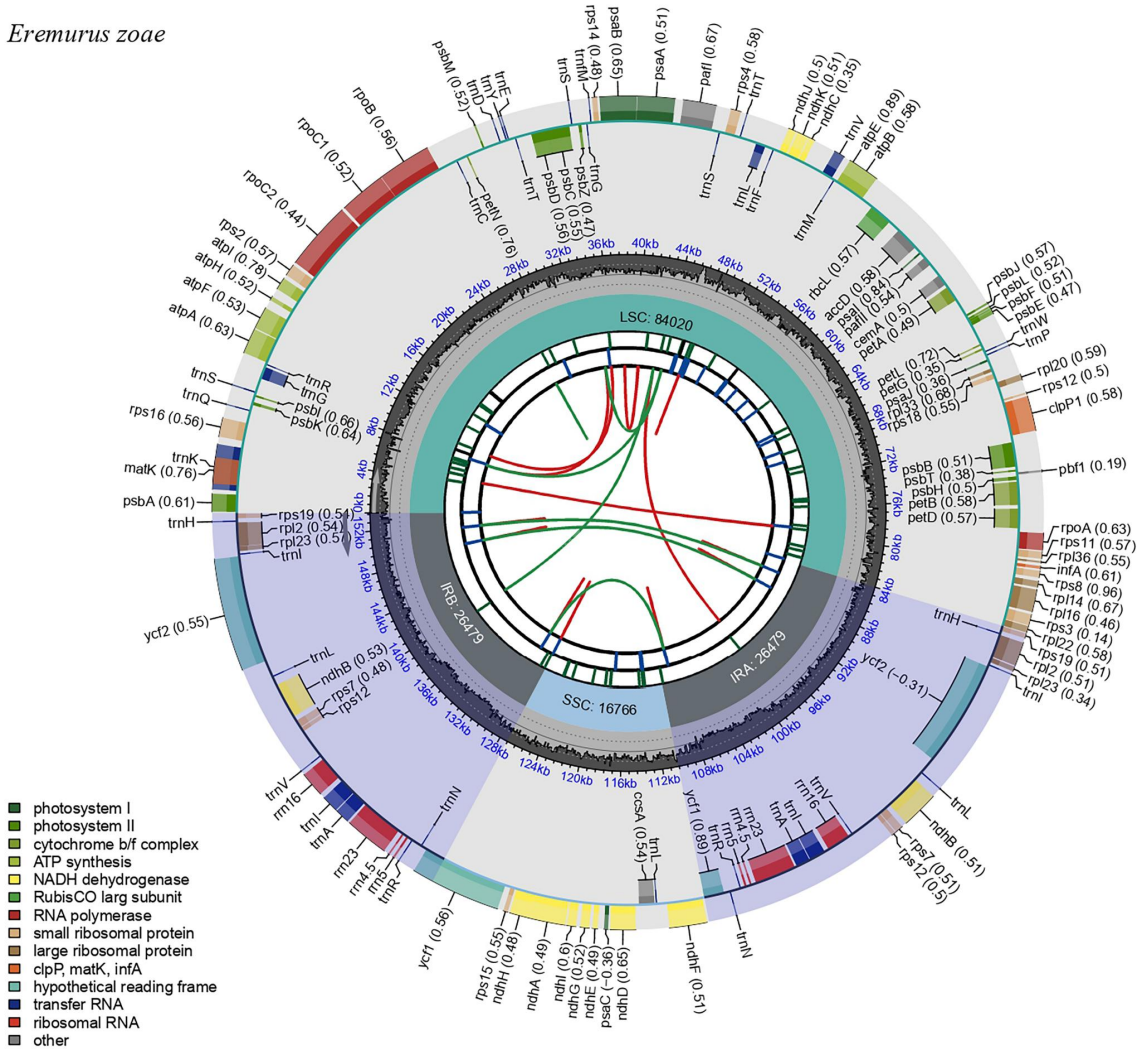
Complete chloroplast genome map of *Eremurus zoae* Vved. drawn using CPGview. The circle map contains six tracks. The first track indicates the repeat distribution. The second track shows the tandem repeats as a blue bar. The third track represents microsatellite sequences in green and yellow. The fourth track shows the LSC, SSC, and IR regions. The fifth track indicates the GC contents, and the sixth track represents genes.

**Figure 3.**
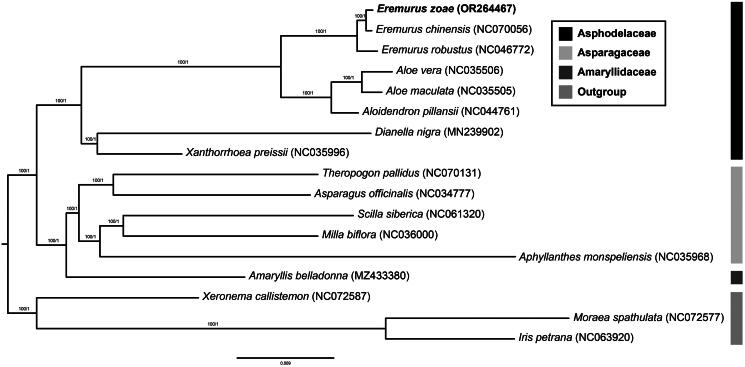
Phylogenetic tree of 17 species with three outgroup taxa (one Xeronemataceae, two Iridaceae) based on 82 protein-coding genes using the ML and BI methods. The numbers above the nodes indicate the bootstrap support values and the Bayesian posterior probabilities. The newly reported chloroplast genome in this study is indicated in red. All sequences used in the analysis are as follows: *Eremurus zoae* (OR264467, this study), *Eremurus chinensis* (NC070056), *Eremurus robustus* (NC046772, Makhmudjanov et al.^2019^), *Aloe vera* (NC035506), *Aloe maculata* (NC035505), *Aloidendron pillansii* (NC044761), *Dianella nigra* (MN239902), *Xanthorrhoea preissii* (NC035996), *Theropogon pallidus* (NC070131), *Asparagus officinalis* (NC034777), *Scilla siberica* (NC061320), *Milla biflora* (NC036000), *Aphyllanthes monspeliensis* (NC035968), *Amaryllis belladonna* (MZ433380), *Xeronema callistemon* (NC072587, Kamra et al.^2023^), *Moraea spathulata* (NC072577, Kamra et al.^2023^), and *Iris petrana* (NC063920, Volis et al.^2022^).

The cp genome of *E. zoae* was 153,744 bp in length, with an overall GC content of 42.9% and an average coverage of 446.60 (Figure 2, Figure S1). The assembled genome has a typical quadripartite structure, including a large single copy (LSC: 84,020 bp), a small single copy (SSC: 16,766 bp), and a pair of inverted repeats (IRa and IRb: 26,479 bp). The cp genome contained 132 genes (86 protein-coding genes, 38 tRNA genes, and 8 rRNA genes), including 20 genes (8 protein-coding genes, 8 tRNA genes, and 4 rRNA genes) duplicated in the IR regions. In total, 11 cis-splicing genes (Figure S2) and one trans-splicing gene, rps12 (Figure S3), were detected. Of the 11 cis-splicing genes, nine (*rps16*, *atpF*, *rpoC1*, *petB*, *petD*, *rpl16*, *rpl2*, *ndhB*, and *ndhA*) contained one intron, and two (*pafl, clpP1*) contained two introns. Gene *rpl32* was missing in some species of subfamily Asphodelaceae, which is related to *Eremurus* zoae (including genus *Eremurus*, *Aloe*, *Aloidendron* in Figure 3). The absence of *rpl32* gene has been recently reported in *Aloe vera* (Xie et al. 2019). Gene losses in plastid genomes might be attributed to the functional transfer to the nucleus or replacement of nuclear genes (Shrestha et al. 2020; Han et al. 2022).

The topologies of the two methods (ML and BI) precisely correspond. Phylogenetic trees strongly supported the idea that Asphodelaceae is a monophyletic group (bootstrap value = 100). Within Asphodelaceae, three subfamilies (Asphodeloideae, Xanthorrhoea, and Hemerocallidoideae) formed distinct clades with high support values. *E. zoae* was the most closely related to *E. chinensis* (Figure 3). Our results are consistent with those of a previous Asparagales phylogenetic study (Chase et al. 2009).

## Conclusions

In this study, we reported the first complete chloroplast genome of *E. zoae*. The plastid genome of *E. zoae* was determined to be 153,744 bp in length, containing a total of 132 genes. A phylogenetic tree reconstructed by concatenated 82 protein-coding genes showed that *Eremurus* formed a monophyletic group. Our tree also revealed that *E. zoae* was most closely related to *E. chinensis*. This result provides useful information regarding the phylogenetic system and evolution of the genus *Eremurus*.

## Supplementary Material

Supplemental Material

## Data Availability

The genome sequence data supporting the findings of this study are openly available in GenBank of NCBI at [https://www.ncbi.nlm.nih.gov] (https://www. ncbi. nlm. nih. gov) under accession no. OR264467. The associated BioProject, SRA, and Bio-Sample numbers are PRJNA996712, SRR25367042, and SAMN36621056, respectively.

## References

[CIT0001] APG IV. 2016. An update of the Angiosperm Phylogeny Group classification for the orders and families of flowering plants: APG IV. Bot J Linn Soc. 181(1):1–20. doi:10.1111/boj.12385.

[CIT0002] Chase MW, Reveal JL, Fay MF. 2009. A subfamilial classification for the expanded asparagalean families Amaryllidaceae, Asparagaceae and Xanthorrhoeaceae. Bot J Linn Soc. 161(2):132–136. doi:10.1111/j.1095-8339.2009.00999.x.

[CIT0003] Cho KS, Yun BK, Yoon YH, Hong SY, Mekapogu M, Kim KH, Yang TJ. 2015. Complete chloroplast genome sequence of tartary buckwheat (*Fagopyrum tataricum*) and comparative analysis with common buckwheat (*F. esculentum*). PLoS One. 10(5):e0125332. doi:10.1371/journal.pone.0125332. 25966355 PMC4428892

[CIT0004] Farhadi F, Avan R, Sahebkar A, Eghbali S. 2023. A comprehensive review on *Eremurus* species: phytochemistry, pharmacology and traditional uses. Phytochem Lett. 53:142–149. doi:10.1016/j.phytol.2022.12.002.

[CIT0005] Fedchenko BA. 1968. Eremurus. In: komarov VL, editor. Flora of the USSR. Vol. 4. Leningrad: botanical Institute of Academy of Science; p. 27–40.

[CIT0007] Guindon S, Dufayard JF, Lefort V, Anisimova M, Hordijk W, Gascuel O. 2010. New algorithms and methods to estimate maximum-likelihood phylogenies: assessing the performance of PhyML 3.0. Syst Biol. 59(3):307–321. doi:10.1093/sysbio/syq010. 20525638

[CIT0008] Han S, Ding H, Bi D, Zhang S, Yi R, Gao J, Yang J, Ye Y, Wu L, Kan X. 2022. Structural Diversities and Phylogenetic Signals in Plastomes of the Early-Divergent Angiosperms: A Case Study in Saxifragales. Plants. 11(24):3544. doi:10.3390/plants11243544. 36559654 PMC9787361

[CIT0009] Hedge I, Wendelbo P. 1963. Notes on the giant Asphodels of Afghanistan. J Roy Hort Soc London. 88(9):402–406.

[CIT0010] Jin JJ, Yu WB, Yang JB, Song Y, dePamphilis CW, Yi TS, Li DZ. 2020. GetOrganelle: a fast and versatile toolkit for accurate *de novo* assembly of organelle genomes. Genome Biol. 21(1):241. doi:10.1186/s13059-020-02154-5. 32912315 PMC7488116

[CIT0011] Kalyaanamoorthy S, Minh BQ, Wong TK, Von Haeseler A, Jermiin LS. 2017. ModelFinder: fast model selection for accurate phylogenetic estimates. Nat Methods. 14(6):587–589. doi:10.1038/nmeth.4285. 28481363 PMC5453245

[CIT0012] Kamenetsky R, Rabinowitch E. 1999. Flowering response of *Eremurus* to post-harvest temperatures. Sci Hortic. 79(1-2):75–86. doi:10.1016/S0304-4238(98)00181-2.

[CIT0013] Kamra K, Jung J, Kim JH. 2023. A phylogenomic study of Iridaceae Juss. based on complete plastid genome sequences. Front Plant Sci. 14:1066708. doi:10.3389/fpls.2023.1066708. 36844099 PMC9948625

[CIT0014] Karaman K, Polat B, Ozturk I, Sagdic O, Ozdemir C. 2011. Volatile compounds and bioactivity of *Eremurus spectabilis* (Ciris), a Turkish wild edible vegetable. J Med Food. 14(10):1238–1243. doi:10.1089/jmf.2010.0262. 21548806

[CIT0015] Katoh K, Standley DM. 2013. MAFFT multiple sequence alignment software version 7: improvements in performance and usability. Mol Biol Evol. 30(4):772–780. doi:10.1093/molbev/mst010. 23329690 PMC3603318

[CIT0016] Kearse M, Moir R, Wilson A, Stones-Havas S, Cheung M, Sturrock S, Buxton S, Cooper A, Markowitz S, Duran C, et al. 2012. Geneious basic: an integrated and extendable desktop software platform for the organization and analysis of sequence data. Bioinformatics. 28(12):1647–1649. doi:10.1093/bioinformatics/bts199. 22543367 PMC3371832

[CIT0017] Kyrgyz Republic. 2006. The Red Data Book of Kyrgyz Republic. 2nd ed. Bishkek: kyrgyz Republic; p. 543.

[CIT0018] Lazkov G, Sennikov AN. 2015. Taxonomic corrections and new records in vascular plants of Kyrgyzstan, 4. Memoranda Societatis Pro Fauna et Flora Fennica. 91:67–83.

[CIT0019] Liu S, Ni Y, Li J, Zhang X, Yang H, Chen H, Liu C. 2023. CPGView: a package for visualizing detailed chloroplast genome structures. Mol Ecol Resour. 23(3):694–704. doi:10.1111/1755-0998.13729. 36587992

[CIT0020] Lowe TM, Chan PP. 2016. TRNAscan-SE online: integrating search and context for analysis of transfer RNA genes. Nucleic Acids Res. 44(W1):W54–W57. doi:10.1093/nar/gkw413. 27174935 PMC4987944

[CIT0021] Makhmudjanov D, Yusupov Z, Abdullaev D, Deng T, Tojibaev K, Sun H. 2019. The complete chloroplast genome of *Eremurus robustus* (Asphodelaceae). Mitochondrial DNA B Resour. 4(2):3366–3367. doi:10.1080/23802359.2019.1674198. 33365996 PMC7707342

[CIT0022] Minh BQ, Nguyen MA, von Haeseler A. 2013. Ultrafast approximation for phylogenetic bootstrap. Mol Biol Evol. 30(5):1188–1195. doi:10.1093/molbev/mst024. 23418397 PMC3670741

[CIT0023] Muhidinov ZK, Bobokalonov JT, Ismoilov IB, Strahan GD, Chau HK, Hotchkiss AT, Liu L. 2020. Characterization of two types of polysaccharides from *Eremurus hissaricus* roots growing in Tajikistan. Food Hydrocolloids. 105:105768. doi:10.1016/j.foodhyd.2020.105768.

[CIT0024] Naderi Safar K, Kazempour Osaloo S, Zarrei M. 2009. Phylogeny of the genus *Eremurus* (Asphodelaceae) based on morphological characters in the Flora Iranica area. Iran J Bot. 15(1):27–35.

[CIT0025] Nguyen LT, Schmidt HA, von Haeseler A, Minh BQ. 2015. IQ-TREE: a fast and effective stochastic algorithm for estimating maximum-likelihood phylogenies. Mol Biol Evol. 32(1):268–274. doi:10.1093/molbev/msu300. 25371430 PMC4271533

[CIT0026] Ronquist F, Teslenko M, van der Mark P, Ayres DL, Darling A, Höhna S, Larget B, Liu L, Suchard MA, Huelsenbeck JP. 2012. MrBayes 3.2: efficient Bayesian phylogenetic inference and model choice across a large model space. Syst Biol. 61(3):539–542. doi:10.1093/sysbio/sys029. 22357727 PMC3329765

[CIT0027] Safar KN, Osaloo SK, Assadi M, Zarrei M, Mozaffar MK. 2014. Phylogenetic analysis of *Eremurus*, *Asphodelus*, and *Asphodeline* (Xanthorrhoeaceae-Asphodeloideae) inferred from plastid trnL-F and nrDNA ITS sequences. Biochem Syst Ecol. 56:32–39. doi:10.1016/j.bse.2014.04.015.

[CIT0028] Shrestha B, Gilbert LE, Ruhlman TA, Jansen RK. 2020. Rampant nuclear transfer and substitutions of plastid genes in *Passiflora*. Genome Biol Evol. 12(8):1313–1329. doi:10.1093/gbe/evaa123. 32539116 PMC7488351

[CIT0029] Song W, Ji C, Chen Z, Cai H, Wu X, Shi C, Wang S. 2022. Comparative analysis the complete chloroplast genomes of nine *Musa* Species: genomic features, comparative analysis, and phylogenetic implications. Front Plant Sci. 13:832884. doi:10.3389/fpls.2022.832884. 35222490 PMC8866658

[CIT0030] Tillich M, Lehwark P, Pellizzer T, Ulbricht-Jones ES, Fischer A, Bock R, Greiner S. 2017. GeSeq–versatile and accurate annotation of organelle genomes. Nucleic Acids Res. 45(W1):W6–W11. doi:10.1093/nar/gkx391. 28486635 PMC5570176

[CIT0031] Volis S, Zhang Y, Deng T, Yusupov Z. 2022. Dark-colored *Oncocyclus* irises in Israel analyzed by AFLP, whole chloroplast genome sequencing and species distribution modeling. Israel J Ecol Evol. 68(1–4):43–53. doi:10.1163/22244662-bja10037.

[CIT0032] Xie DF, Yu HX, Price M, Xie C, Deng YQ, Chen JP, Yu Y, Zhou SD, He XJ. 2019. Phylogeny of Chinese *Allium* species in section *Daghestanica* and adaptive evolution of *Allium* (Amaryllidaceae, Allioideae) species revealed by the chloroplast complete genome. Front Plant Sci. 10:460. doi:10.3389/fpls.2019.00460. 31114591 PMC6503222

[CIT0033] Xinqi C, Songyun L, Jiemei X, Tamura MN. 2000. Liliaceae. Flora of China. In: Wu ZY, Raven PH, editors. Flora of China. Vol 24. Beijing and St. Louis (MO): Science Press and Missouri Botanical Garden Press. p. 73–263.

[CIT0034] Zhang D, Gao F, Jakovlić I, Zou H, Zhang J, Li WX, Wang GT. 2020. PhyloSuite: an integrated and scalable desktop platform for streamlined molecular sequence data management and evolutionary phylogenetics studies. Mol Ecol Resour. 20(1):348–355. doi:10.1111/1755-0998.13096. 31599058

[CIT0035] Zhu Y, Xu C-h, Huang J, Li G-y, Zhou Q, Liu X-H, Sun S-q, Wang J-h 2013. Rapid discrimination of three Uighur medicine of *Eremurus* by FT-IR combined with 2DCOS-IR. J Mol Struct. 1069:96–102. doi:10.1016/j.molstruc.2013.11.054.

