# Supplementary material for: The complete chloroplast genome of Eremurus zoae Vved. (Asphodelaceae), an endemic species of Kyrgyz Republic
Source: Mitochondrial DNA B Resour. 2024 Apr 3;9(4):437–41. doi: 10.1080/23802359.2024.2336003 (PMC10993749; doi:10.1080/23802359.2024.2336003)
Supplement: Supplemental Material [file TMDN_A_2336003_SM6882.docx]

**Supplemental material**

**Figure S1.** Overall coverage depth of the chloroplast genome assembly of *Eremurus zoae*.

**Figure S2.** Schematic map of the cis-splicing genes in the chloroplast genome of *Eremurus zoae*.

**Figure S3** Schematic map of the trans-spliced genes in the chloroplast genome of *Eremurus zoae*.


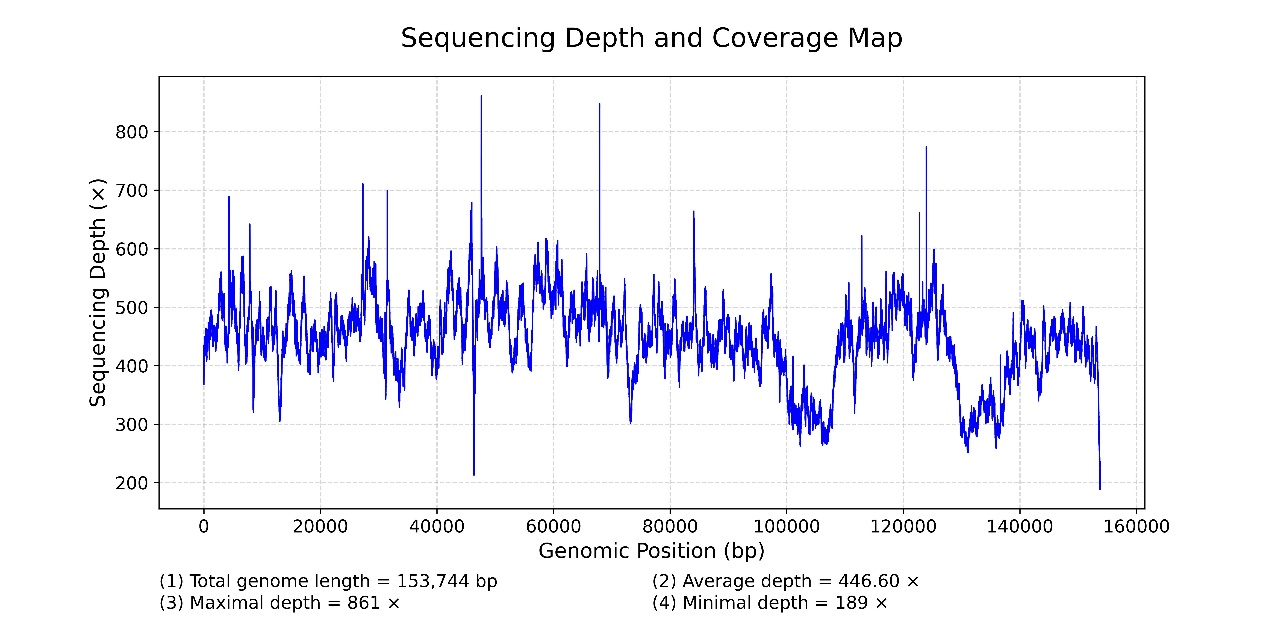
**Figure S1.** Overall coverage depth of the chloroplast genome assembly of *Eremurus zoae*.

**
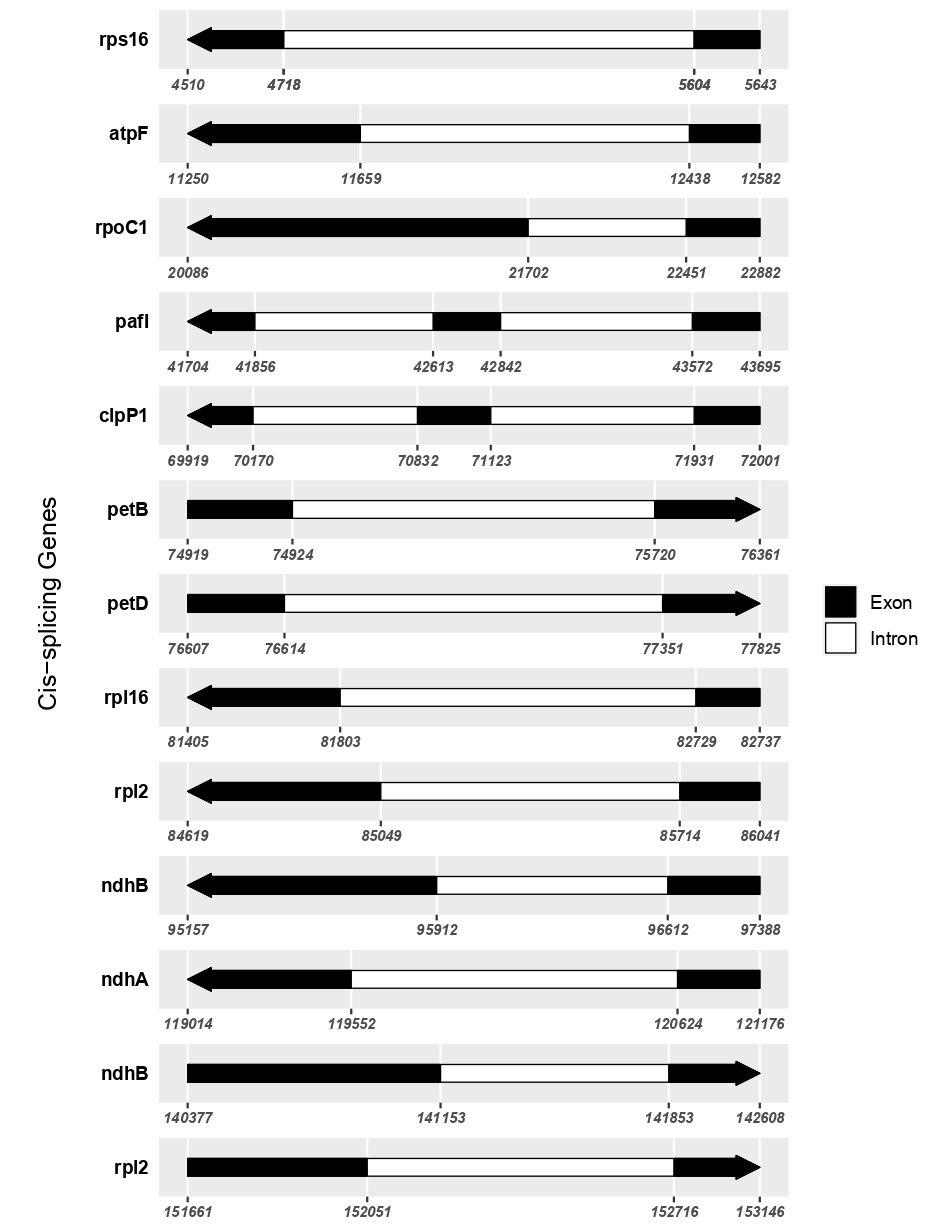
 Figure S2.** Schematic map of the cis-splicing genes in the chloroplast genome of *Eremurus zoae*.


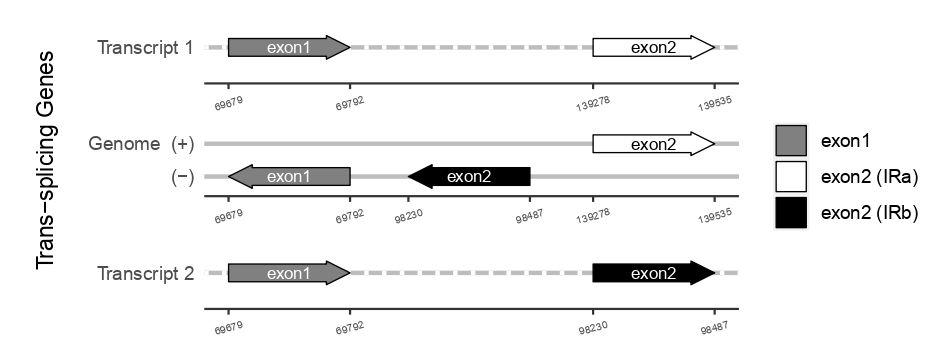


**Figure S3.** Schematic map of the trans-spliced genes in the chloroplast genome of *Eremurus zoae*.
